# The impact of short-term intensive fasting on physical health and gut microbiota in obese individuals

**DOI:** 10.3389/fnut.2026.1873000

**Published:** 2026-08-28

**Authors:** Feng Ji, Shiya Wang, Li Wang, Yixuan Zhao, Jiabin Zhong, Ruohan Wang, Jianing Qu, Yunhang Lu, Na Yuan, Qing Zhang

**Affiliations:** 1 School of Physical Education and Sports Science, Soochow University, Suzhou, China; 2 Changzhou Tourism and Commerce Higher Vocational School, Changzhou, Jiangsu, China; 3 Department of Community Nursing, Soochow University, Suzhou, China; 4 Hematology Center of Cyrus Tang Medical Institute, Soochow University, Suzhou, China; 5 Institute of Health Futures, Soochow University, Suzhou, China

**Keywords:** cardiovascular health, gut microbiome, obesity, physical health, short-term intensive fasting

## Abstract

**Objectives:**

This study aimed to characterize how a 7-day complete water-only fast followed by structured refeeding was associated with changes in body weight, hydration-dependent bioelectrical-impedance estimates, physical test performance, cardiovascular indicators, and gut microbial ecology in adults with obesity and normal-weight controls.

**Methods:**

Seventy-two eligible volunteers formed prespecified BMI sampling strata (normal weight, *n* = 36; obesity, *n* = 36), from which 14 participants were randomly selected within each stratum before the intervention and before outcome collection (*N* = 28). All selected participants completed a supervised 7-day water-only fast followed by a 7-day structured refeeding period. Physical indicators were assessed pre-fasting, post-fasting, and post-refeeding and analyzed using two-factor mixed-design repeated-measures models; gut microbiota (16S rRNA) was profiled pre- and post-fasting.

**Results:**

BMI-group-by-time interactions were significant for body weight and BMI (both *p* < 0.001) but not for BIA-derived fat mass, body-fat percentage, or muscle mass (all *p* > 0.05). Dynamometer-recorded grip, knee-flexion, and knee-extension scores increased over time (all time-effect *p* < 0.001), but none showed a BMI-group-by-time interaction (all *p* ≥ 0.340). Gut microbial alpha diversity remained largely stable apart from an FDR-significant ACE-index reduction in the normal-weight group (*q* = 0.021); most taxonomic findings did not survive FDR correction, and beta-diversity analysis showed no community-level convergence between groups.

**Conclusion:**

Supervised seven-day water-only fasting followed by structured refeeding was associated with reduced body weight, particularly in participants with obesity. Changes in body composition were BIA-derived estimates, while higher dynamometer scores represented measured test performance that may have been influenced by familiarization. Gut-microbiota alpha diversity was largely stable, with exploratory compositional changes supporting further investigation in controlled studies.

## 1 Introduction

Obesity is a chronic metabolic disorder marked by excessive adipose accumulation and disrupted energy homeostasis (1), and it remains one of the most pressing global health challenges (2). Its pathogenesis, however, extends beyond simple energy imbalance: host metabolism and the gut microbiota engage in continuous, bidirectional crosstalk that shapes adiposity (3, 4). Gut dysbiosis can promote obesity through increased energy harvest (5), perturbed lipid metabolism, impaired intestinal barrier function, chronic low-grade inflammation, and altered microbial metabolite signaling (6). In particular, an altered *Firmicutes*-to-*Bacteroidetes* ratio, together with dysregulated production of short-chain fatty acids, bile acids, and lipopolysaccharides, has emerged as a modifiable driver of obesity (7–12). Dietary interventions that recalibrate the gut microenvironment therefore offer an attractive non-pharmacological route to obesity management (13).

Among dietary strategies, intermittent fasting (IF)—including alternate-day fasting, time-restricted feeding, and the 5:2 regimen—promotes weight loss (14, 15), enhances insulin sensitivity (16, 17), and improves cardiovascular biomarkers (18). Yet most IF protocols preserve some exogenous nutrient intake through partial caloric restriction or daily eating windows, so the physiological adaptations they evoke are gradual and modest. What happens when nutrient supply ceases entirely for several consecutive days—and how the gut ecosystem responds to such complete substrate withdrawal—remains poorly resolved.

Short-term intensive fasting, a regimen rooted in traditional Chinese health practice, provides a model to address this question. It comprises consecutive days of complete water-only fasting (0 kcal/day) followed by structured, progressive refeeding, often coupled with mind–body components such as relaxation, meditation, and gentle movement (19–21). The complete cessation of calories distinguishes it physiologically from conventional IF: glycogen depletion and nutrient absence drive a metabolic switch from glucose oxidation toward fatty-acid oxidation and ketogenesis, while the abrupt loss of luminal substrates is expected to impose a distinct selective pressure on gut microbial ecology (22, 23). Supervised studies have reported immune (19) and cardiovascular (24) benefits and a large-scale chart review of 768 cases has confirmed that medically supervised water-only fasting carries a low incidence of serious adverse events (25). When implemented in accordance with established clinical fasting guidelines (26), this protocol can be safely conducted in a medical setting. But whether a seven-day water-only fast induces concordant adaptations across body composition, physical function, and gut microbiota—and whether these responses differ between individuals with obesity and normal-weight controls—has not been systematically examined.

To address these gaps, we conducted a prospective pilot study of a seven-day supervised water-only fast followed by a seven-day structured refeeding period in adults with obesity and normal-weight controls. We tracked body weight, BIA-derived body-composition estimates, dynamometer-based test performance, and cardiovascular indicators at three time points (pre-fasting, post-fasting, and post-refeeding) and profiled 16S rRNA gut microbiota diversity and composition before and immediately after the fast. By integrating physiological and microbiome readouts while explicitly recognizing the limitations of hydration-dependent BIA and repeated performance testing, this study provides exploratory evidence on acute responses to complete energy deprivation.

## 2 Materials and methods

### 2.1 Study design

This study was designed as a prospective, single-arm, pre-post interventional pilot study. It was approved by the Ethics Review Committee of Soochow University (Approval No. ECSU-2019000153) and registered with the Chinese Clinical Trial Register (Registration No. ChiCTR1900027451).^1^ All participants provided written informed consent after receiving a full explanation of the procedures and voluntarily agreed to participate.

ChiCTR1900027451 covered a broader research program investigating physiological responses to supervised short-term intensive fasting. The participants included in the present study constituted a separately recruited cohort and did not overlap with participants included in previous publications associated with this registration (19, 24, 27, 28). Although independently recruited cohorts underwent fasting within the same centrally managed research program and shared operational infrastructure, participant enrollment, study identification, outcome assessment, sample collection, data management, and statistical analysis were conducted separately. No participant-level data, biological samples, outcomes, or statistical results reported in the present manuscript have been published previously. The present cohort intervention was conducted from 11 November 2019 to 27 November 2019, comprising a 3-day standardized dietary lead-in, a 7-day water-only fasting intervention, and a 7-day structured refeeding period.

### 2.2 Participants

Seventy-two volunteers met the eligibility criteria and formed a prespecified sampling frame comprising 36 normal-weight and 36 obese individuals. Because analytical capacity for comprehensive physiological assessment and 16S rRNA sequencing was prespecified at 14 participants per BMI stratum, a computer-generated stratified random selection was performed before the intervention and before any outcome data were collected. Consequently, 28 participants entered the intervention and analytical cohort. The remaining 44 eligible volunteers did not enter the intervention or undergo outcome assessment and were therefore not study withdrawals or post-enrollment exclusions. Participants were classified according to the BMI criteria of the “Guidelines for the Prevention and Control of Overweight and Obesity in Chinese Adults” (29). Sex-stratified summaries were treated as exploratory because the resulting subgroups were small and were not powered for formal sex-by-time interaction testing.

Inclusion criteria: (1) Age range: 18–65 years; gender unrestricted; (2) Normal weight group subjects: 18.5 kg/m^2^ ≤ BMI < 24 kg/m^2^; Obese group subjects: BMI ≥ 28 kg/m^2^, BMI = weight (kg) ÷ height (m)^2^; (3) Prior to enrollment, participants were thoroughly informed about the seven-day intensive fasting protocol, provided written informed consent, demonstrated satisfactory adherence to all scheduled assessments, and confirmed their willingness to comply with the experimental procedures.

Exclusion criteria: (1) Pregnant and lactating women; (2) Participants with a history of mental illness, severe organic diseases, or infectious diseases; (3) Participants with severe hypoglycemia, hypotension, or impaired liver function; (4) Participants with gastrointestinal diseases, such as chronic gastritis, severe diarrhea, or constipation, etc.; (5) Participants who used antibiotics, probiotics, or medications affecting metabolism (e.g., hypoglycemics, lipid-lowering drugs) within 1 month prior to the study.

Withdrawal criteria: (1) Poor compliance, unwillingness to continue the experiment, or non-cooperation with scheduled assessments; (2) Occurrence of apparent discomfort during the experiment; (3) Failure to perform prescribed exercises or resumption of normal diet during the intensive fasting period.

### 2.3 Short-term intensive fasting program

Intensive fasting was implemented as previously described (24). Prior to intensive fasting, researchers presented the entire intensive fasting process and precautions to the participants through photos or videos to ensure adequate mental preparation. The intensive fasting program was guided by professional intensive fasting instructors and three teaching assistants, and supervised by a comprehensive team of certified professionals in the fields of medicine, life sciences, health management, and nursing. Prior to the experiment, the research team rehearsed the specific procedures to ensure safety, consistency, and reproducibility. Additionally, participants’ physical health was systematically assessed to confirm fitness for fasting. An emergency rescue kit was provided to ensure timely treatment during the critical early fasting period (Days 1–4).

The intensive fasting intervention lasted for 1 week. During the 3-day lead-in period prior to the water-only fasting, participants consumed a standardized, energy-restricted plant-based diet (~1,200–1,500 kcal/day; ~65% carbohydrates, 20% fats, and 15% proteins) devoid of meat, alcohol, caffeine, and high-fat processed foods. Dietary compliance was verified daily via electronic food logs submitted through a mobile messaging application and reviewed by a certified dietitian. During the seven-day intensive fasting period, participants were centrally accommodated at the Kempinski Hotel in Suzhou for the first 4 days, then continued intensive fasting at home for the last 3 days, consuming only moderate amounts of water and no other food or beverages. Water intake was not restricted because prolonged fasting leads to changes in the body’s energy metabolism, typically increasing fat metabolism during this phase; since the breakdown of fat requires more water than the breakdown of sugar, slightly increased water intake facilitates metabolic processes (30).

To monitor adherence to the water-only fasting protocol, participants were fully supervised during the initial 4 days at our facility and were required to maintain standardized electronic fasting journals throughout the 7-day period. During the final 3 days completed at home, compliance was verified via mandatory daily remote check-ins (video and telephone consultations) conducted by supervising medical staff.

To facilitate adherence to the fasting protocol, participants engaged in a standardized mind–body relaxation program during the facility-based phase (Days 1–4), comprising 45-min sessions of diaphragmatic breathing meditation followed by gentle stretching and self-myofascial release techniques (20). Participants were also instructed in cognitive strategies for managing hunger sensations, including mindful acceptance and behavioral substitution practices (e.g., self-dialogue, swallowing exercises). Throughout the intervention, participants were advised to maintain a calm demeanor and avoid intense physical exertion or emotional distress; on-site counseling was readily available for participants experiencing anxiety or other adverse psychological reactions. Post-fasting, participants adhered to a structured refeeding protocol designed by nutritionists (detailed below), focusing on gradual caloric reintroduction, micronutrient balance, and avoidance of high-fat/sugar foods. After resuming normal diet, participants were instructed to maintain balanced eating patterns and healthy lifestyle habits.

During the fasting period, participants were advised to arrange their work schedules to avoid conventional mealtimes (to reduce conditioned eating reflexes). Physical labor or intense exercise was avoided throughout the entire intensive fasting project. The water-only fasting was discontinued when any participant decided to withdraw or when health management professionals deemed it medically necessary. Data measurement and collection were centralized at the hotel before the start of intensive fasting, after the seven-day intensive fasting, and after the seven-day reintroduction diet.

### 2.4 Refeeding plan

General notes: all participants received standardized measuring tools to ensure consistency. The volume of the bowls was calibrated to 250 mL. Salt intake was restricted to <3 g/day and oil to <10 g/day, in accordance with WHO guidelines (31).

1. Day 1: liquid reintroduction

Participants consumed 5–6 meals daily, each consisting of 200 mL of millet porridge prepared by boiling 60 g of dry millet in 400 mL of water. Up to 5 mL of olive oil was permitted per meal. Water intake was maintained at 35 mL/kg of body weight per day.

1. Day 2: semi-liquid transition

Participants had three primary meals, each comprising 200 mL of millet porridge or 250 mL of vegetable soup (pumpkin, carrot). Additionally, 2–3 servings of 30 g infant rice cereal, reconstituted with 100 mL of water per serving, were provided as snacks. A chewing protocol requiring 20–25 chews per bite was implemented to minimize gastrointestinal load.

1. Day 3: soft solid introduction

On Day 3, breakfast consisted of 200 mL of millet porridge. For lunch and dinner, participants consumed 50 g of cooked noodles accompanied by 100 g of finely chopped vegetables (spinach, zucchini). Seasoning was limited to ≤1 g of salt per meal. Intake was terminated when 50% of the estimated daily caloric needs were met (approximately 1,000 kcal for females and 1,200 kcal for males).

1. Day 4: expanded texture

On Day 4, breakfast options included 200 mL of rice porridge or 50 g of noodles. Lunch and dinner consisted of 80 g of soft rice paired with 150 g of stir-fried vegetables, using ≤3 g of oil per serving. A 100 g serving of low-fiber fruits, such as watermelon or peeled pear, was also included.

1. Day 5: protein integration

Breakfast options were 200 mL of rice porridge, 50 g of noodles, or 2 boiled eggs (approximately 100 g). Lunch and dinner comprised 100 g of rice, 150 g of vegetables, and 50 g of tofu, prepared by steaming or boiling. Intake was halted at 70% of the estimated daily caloric requirements.

1. Day 6: near-normal diet

Participants resumed a regular diet, excluding fried foods and meat. Portion control was emphasized, with carbohydrates limited to 100–120 g of cooked grains per meal and vegetables to 150–200 g per meal. Meals with >10 g of fat continued to be avoided.

1. Day 7: full refeeding

100 g of steamed white fish (e.g., tilapia) was introduced into the daily diet. High-fiber foods (>5 g fiber per serving, such as whole grains and legumes) were avoided. The daily caloric intake was increased by 10–15% every 2 days until maintenance levels were achieved.

During the refeeding period after intensive fasting, dietary adjustment is crucial and must be strictly followed to avoid damaging the stomach and intestines.

### 2.5 Precautions

1. Certified fasting instructors, who possess expertise in nutrition, physiology, and mindfulness practices, and have extensive experience in conducting intensive fasting programs, supervised the intervention.
2. Participants were required to practice breathing exercises daily and communicate promptly with staff if feeling unwell.
3. Intense physical activities were prohibited during the intensive fasting period. Participants were advised to maintain a calm mindset and avoid extreme emotional fluctuations.
4. Transient halitosis and body odor may be observed during the later days of intensive fasting; these symptoms are self-limiting and resolve upon resuming normal diet.
5. Refeeding errors—the abrupt consumption of high-calorie, high-fat, or high-fiber foods—can provoke gastrointestinal distress. Intake should progress from liquids to solids, from small to larger portions, and from vegetarian items toward meat, gradually reaching a balanced diet of 55–60% carbohydrate, 15–20% protein, and 25–30% fat, in line with the Chinese Dietary Guidelines (32).

### 2.6 Outcome measurement

1. Physical health indicators were measured at three time points: before fasting (0 w), immediately after the seven-day fast (1 w), and 1 week after refeeding (2 w). All 14 normal-weight participants completed all assessments. In the obesity group, one participant had no body-composition or dynamometer data, while a different participant had no cardiovascular data. Accordingly, each physical-outcome model included 14 normal-weight and 13 obesity-group participants, but the identity of the participant with missing data differed by outcome domain.

Body composition and phase angle (PhA): body composition and PhA were measured with a multifrequency bioimpedance analyzer (InBody 770). Participants emptied their bladders beforehand and stood barefoot on the platform, forefeet and heels on the electrodes, holding the handles with thumbs on the contacts and arms slightly abducted, remaining still and silent throughout the measurement. The analyzer derives weight and BMI and partitions fat and muscle mass from their differing electrical resistance (33, 34). PhA at 50 kHz—a frequency-independent parameter reflecting cellular membrane integrity, body cell mass, and hydration status—was recorded simultaneously.

Waist-to-Hip Ratio (WHR): waist and hip circumferences were measured with a soft tape measure. Participants stood relaxed in training attire, with feet hip-width apart for waist measurement, measuring the circumference around the navel; for hip measurement, feet were together, and the circumference was measured at the most prominent part of the gluteal muscles.

Muscle strength: several major muscle groups commonly used in daily life were measured, including grip strength and the strength of knee extension and flexion. Grip strength was measured using a hand dynamometer (Jamar Plus, model 563213, USA). Participants stood in a standard position with their hands naturally hanging down, using their dominant hand for two tests, and the best score was taken. Knee extension and flexion strength were measured using a Micro FET 3 portable muscle strength and joint range of motion tester (Hoggan, model 120382, USA). For flexion strength, participants lay face down on a yoga mat with the device fitted with a bend sensor pad behind the ankle, slowly bending the knee to obtain the strength value. For extension strength, participants sat on a high stool with their feet not touching the ground, with the device’s bend sensor pad in front of the ankle, slowly extending the knee to read the strength value.

Blood pressure, heart rate fluctuations, and vascular stiffness indicators: To ensure the safety of the intensive fasting program, this study monitored blood pressure and heart rate fluctuations. Additionally, vascular stiffness was evaluated using two key indicators: the augmentation index value corrected for a heart rate of 75 beats per minute (AIX@75 bpm) and pulse wave velocity (PWV). The AIX reflects the elasticity of peripheral small arteries and the degree of arteriosclerosis, with values gradually increasing with age, normally ranging from −5 to 40. Values less than −5 can increase the risk of myocardial ischemia and left ventricular hypertrophy caused by increased afterload, while values greater than 40 can increase the risk of stroke; PWV is the propagation speed of pulse waves in arterial segments, reflecting arterial elasticity and compliance, with increased values indicating increased arterial stiffness, and values greater than 10 m/s suggesting a certain degree of aortic sclerosis (35–37). These indicators were measured using a pulse wave analysis device (Mobil-O-Graph PWA, Germany). Before measurement, participants were required to calm down and avoid vigorous exercise. During measurement, participants sat on a stool with their left forearm on the table at heart level, palm up, with the device’s cuff tied around the middle of the upper arm with a tightness that allows one finger to fit inside, starting the device, and saving the data after the measurement was completed.

1. Gut microbiome: all 28 participants provided fecal samples before intensive fasting (0 w) and 1 week after intensive fasting (1 w). Before sample collection, participants received standardized instructions regarding fecal collection procedures, including avoidance of contamination with urine or other substances. Fecal collection tubes were provided, and samples were collected, labeled, packaged, and immediately stored at −80 °C until further analysis.

Fecal DNA extraction, amplification, and sequencing were performed by Microbase Biotechnology Co., Ltd. Microbial genomic DNA was extracted from fecal samples, followed by PCR amplification and sequencing using the MiSeq high-throughput sequencing platform. Raw sequencing reads were subjected to quality control and filtering, and the optimized sequences were clustered into operational taxonomic units (OTUs) at a 97% similarity threshold. Taxonomic annotation was performed based on OTU representative sequences. Genera with relative abundances ≤1% were grouped into the category “Others” for visualization and statistical presentation.

Gut microbial diversity was evaluated using both alpha diversity (α-diversity) and beta diversity (β-diversity) analyses. Alpha diversity was assessed using the Chao1, ACE, Shannon, and Simpson indices (38). The Chao1 and ACE indices were used to estimate microbial richness, with higher values indicating greater richness, whereas the Shannon and Simpson indices were used to evaluate community diversity, with higher Shannon values and lower Simpson values representing greater diversity (39–41). β-diversity was assessed to evaluate differences in overall microbial community composition among samples (42). Bray–Curtis dissimilarity matrices were calculated based on genus-level relative abundance profiles, and principal coordinates analysis (PCoA) was performed for visualization of microbial community distribution patterns. Differences in microbial community structure among groups and time points were evaluated based on β-diversity analysis. The present study analyzed changes in gut microbial α- and β-diversity before and after intensive fasting, as well as alterations in microbial composition at the phylum, order, family, and genus levels.

### 2.7 Statistical method

Microbiome analyses were performed using IBM SPSS Statistics 26.0 (IBM Corp., Armonk, NY, USA) and the procedures described below. Physical-health analyses were independently rerun in Python 3.13.9 (NumPy 2.3.5, pandas 2.3.3, SciPy 1.16.3, and statsmodels 0.14.5). All tests were two-sided, with *p* < 0.05 considered statistically significant. Data are reported as mean ± standard deviation (SD), and effect sizes are reported as partial eta squared (*η_p_*^2^) where applicable.

1. Physical health indicators: for each outcome, a two-factor mixed-design repeated-measures analysis of variance was fitted with time (pre-fasting [0 w], post-fasting [1 w], and post-refeeding [2 w]) as the within-participant factor, BMI group (normal weight and obesity) as the between-participant factor, and the BMI-group-by-time interaction. Differential response between BMI groups was evaluated only from the interaction term; a significant change in one group and a nonsignificant change in the other was not interpreted as evidence that responses differed. Greenhouse–Geisser-adjusted degrees of freedom and *p* values were used for the time and interaction effects to reduce reliance on the sphericity assumption. Where the omnibus time effect was significant, prespecified within-group paired contrasts (0 w vs. 1 w, 0 w vs. 2 w, and 1 w vs. 2 w) were examined with Holm adjustment within each outcome and BMI group. Analyses used complete observations separately for each outcome domain (normal weight, *n* = 14; obesity, *n* = 13), and partial eta squared (*η_p_*^2^) quantified effect size.
2. Gut microbiota — alpha diversity and taxonomic composition: within-group changes (0 w vs. 1 w) in α-diversity indices (Chao1, ACE, Shannon, Simpson) and taxonomic relative abundances (phylum through species levels) were assessed using Wilcoxon signed-rank tests. Between-group comparisons at each time point used Mann–Whitney *U* test (non-normal) or independent-samples t-test (normal, verified by Shapiro–Wilk). Benjamini-Hochberg false discovery rate (FDR) correction was applied within each comparison family; taxa with nominal *p* < 0.05 are reported as exploratory findings, while those surviving FDR correction (*q* < 0.05) are emphasized as robust results.
3. Gut microbiota — beta diversity: β-diversity was assessed using Bray–Curtis dissimilarity matrices computed from genus-level relative abundance data. Ordination via PCoA. PERMANOVA (999 permutations) tested for between-group differences at baseline (0 w) and post-fasting (1 w), as well as within-group compositional change (0 w → 1 w). Given that only relative abundance data (rather than raw read counts) were available, Bray–Curtis dissimilarity was computed on relative abundances, an accepted approach for compositional data.
4. Sex-stratified summaries: because the sex-by-BMI subgroups contained only 5–8 participants and were not powered for a sex-by-time-by-BMI interaction, sex-stratified results are presented descriptively as exploratory supplementary analyses. Differences in whether a within-subgroup *p* value crossed 0.05 were not interpreted as evidence of different responses by sex.
5. Sample size justification: no formal *a priori* sample-size calculation was performed because this was an exploratory, capacity-limited pilot study. Recruitment created two BMI-defined sampling strata of 36 eligible volunteers each. Analytical capacity was prespecified at 14 participants per stratum because comprehensive physiological phenotyping and 16S rRNA sequencing were resource intensive; random selection occurred within each stratum before the intervention and before outcome collection. The analytical sample was therefore determined by prespecified capacity rather than an assumed effect size. No *post hoc* achieved-power calculation was used to interpret results; exact *p* values and effect sizes are reported, and nonsignificant findings are not treated as evidence of equivalence. The study was not designed to detect modest BMI-group-by-time or sex-related interactions. For physical outcomes, one obesity-group participant lacked body-composition and dynamometer data and a different obesity-group participant lacked cardiovascular data, resulting in *n* = 13 for each corresponding model.

Graphs were plotted using GraphPad Prism 9 and the CNSknowall platform.^2^

## 3 Results

### 3.1 Participant flow and baseline characteristics

**Figure 1:**
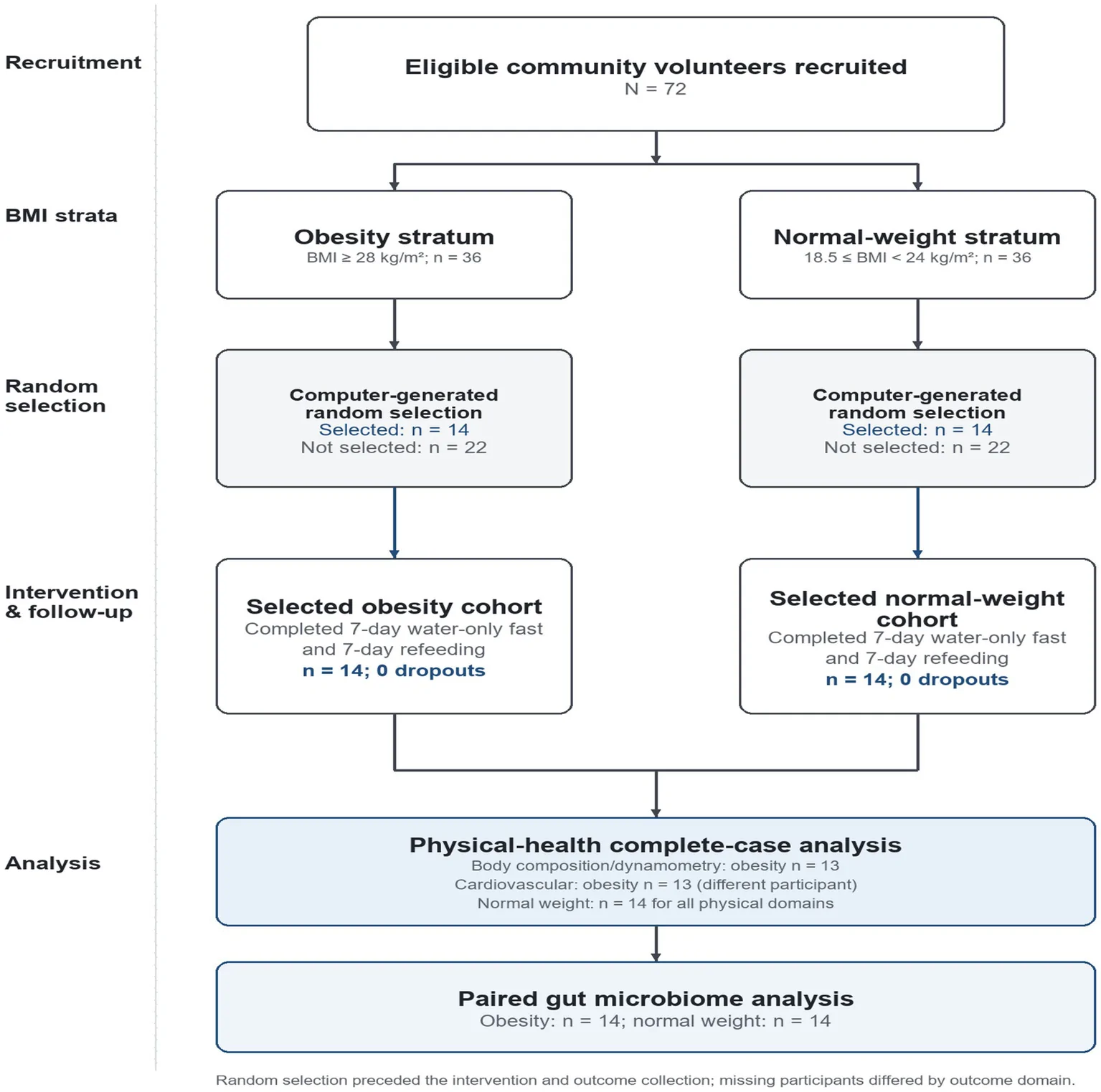
Participant flow diagram. Seventy-two eligible volunteers formed prespecified BMI sampling strata (normal weight, *n* = 36; obesity, *n* = 36). Before the intervention and before outcome collection, 14 participants were randomly selected within each stratum for the analytical cohort (*N* = 28); the remaining 22 volunteers per stratum did not enter the intervention and were not withdrawals or post-enrollment exclusions. All selected participants completed the 7-day water-only fast and 7-day refeeding periods. One obesity-group participant lacked body-composition and dynamometer data, and a different obesity-group participant lacked cardiovascular data (*n* = 13 for each domain); all selected participants contributed paired microbiome data.

Seventy-two eligible volunteers formed two prespecified BMI sampling strata (normal weight, *n* = 36; obesity, *n* = 36). Before the intervention and before outcome collection, 14 participants were randomly selected within each stratum for the analytical cohort (*N* = 28); the remaining 44 eligible volunteers did not enter the intervention or undergo outcome assessment. All 28 selected participants completed the 7-day water-only fast and 7-day refeeding periods without dropout or clinical withdrawal (Figure 1). In the obesity group, one participant lacked body-composition and dynamometer data, while a different participant lacked cardiovascular data; consequently, each physical-outcome domain included *n* = 13 obesity-group participants. All 28 selected participants provided paired fecal samples for microbiome analyses.

The normal-weight group averaged 40.64 ± 6.36 years (8 women, 6 men) and the obese group 49.00 ± 11.18 years (6 women, 8 men). To ensure baseline comparability and minimize metabolic confounding, screening questionnaires confirmed that all participants habitually consumed a standard omnivorous diet, led sedentary-to-moderately-active lifestyles, and reported no extreme dietary regimens or structured high-intensity exercise. Per the exclusion criteria, the cohort was free of active major comorbidities (cardiovascular, hepatic, renal, or gastrointestinal disorders) and mental illness, and no participant had used antibiotics, probiotics, or metabolism-affecting medications within 1 month before enrollment.

### 3.2 Changes in body weight and BIA-derived body-composition estimates

The two-factor repeated-measures model showed significant overall time effects for body weight, BMI, waist-to-hip ratio, and all InBody 770 outputs (all Greenhouse–Geisser-adjusted *p* < 0.011). In the normal-weight group, body weight was 3.64 kg lower immediately after fasting and 2.70 kg lower after refeeding. The device also reported lower BIA-derived fat-mass estimates (1.55 kg at 1 w; 2.04 kg at 2 w), subcutaneous-fat estimates (1.41 kg; 1.65 kg), and muscle-mass estimates (2.41 kg; 1.11 kg) relative to baseline.

In the obesity group, body weight was 8.20 kg lower immediately after fasting and 8.63 kg lower after refeeding. At 1 w, the InBody 770 reported decreases of 3.28 kg in estimated fat mass, 2.37 percentage points in estimated body-fat percentage, 2.72 kg in estimated subcutaneous fat, 0.55 in the estimated visceral-fat level, and 1.72 kg in estimated muscle mass. The corresponding BIA-derived estimates remained numerically below baseline at 2 w.

BMI-group-by-time interactions were significant for body weight, *F*_(1.60, 40.12)_ = 9.71, *p* < 0.001, *η_p_*^2^ = 0.280, and BMI, *F*_(1.63, 40.84)_ = 10.13, *p* < 0.001, *η_p_*^2^ = 0.288, indicating different trajectories between the BMI groups. The interaction was nominally significant for the BIA-derived visceral-fat level, *F*_(1.61, 40.24)_ = 3.49, *p* = 0.049, *η_p_*^2^ = 0.123, but not for waist-to-hip ratio, BIA-derived fat mass, body-fat percentage, subcutaneous fat, or muscle mass (all *p* ≥ 0.067). Because multiple exploratory outcomes were tested and the visceral-fat result was borderline, it should be interpreted cautiously. The BIA outcomes remain hydration-sensitive estimates rather than direct tissue measurements (Table 1).

**Table 1:** Body weight and BIA-derived body-composition estimates during fasting and refeeding.

Indicator	Normal weight group (*n* = 14)			Obesity group (*n* = 13)		
	Pre-fasting (0 w)	Post-fasting (1 w)	Refeeding (2 w)	Pre-fasting (0 w)	Post-fasting (1 w)	Refeeding (2 w)
Weight (kg)	60.66 ± 8.21	57.02 ± 7.23**	57.97 ± 7.51**	81.34 ± 9.37	73.14 ± 11.21**	72.71 ± 10.55**
BMI (kg/m^2^)	21.86 ± 1.41	20.71 ± 1.20^**^	20.90 ± 1.33**	28.68 ± 0.82	25.76 ± 2.28^**^	25.62 ± 2.08**
WHR	0.88 ± 0.05	0.88 ± 0.04	0.86 ± 0.04	0.95 ± 0.03	0.96 ± 0.03	0.95 ± 0.03
BIA-derived fat mass (kg)	14.32 ± 4.35	12.77 ± 4.14**	12.29 ± 3.95**	24.32 ± 5.44	21.05 ± 5.03**	20.95 ± 5.02**
BIA-derived body fat percentage (%)	24.00 ± 8.16	22.98 ± 8.39	21.72 ± 7.75**	31.38 ± 6.68	29.01 ± 6.44**	28.94 ± 6.32**
BIA-derived subcutaneous fat (kg)	12.59 ± 3.78	11.18 ± 3.58**	10.94 ± 3.51**	19.78 ± 4.29	17.05 ± 3.91**	17.12 ± 4.08**
BIA-derived visceral fat level	1.73 ± 0.64	1.59 ± 0.61	1.34 ± 0.52**	4.55 ± 1.36	3.99 ± 1.26**	3.83 ± 1.12**
BIA-derived muscle mass (kg)	44.21 ± 9.12	41.79 ± 8.93^**^	43.10 ± 8.86^*^	50.79 ± 10.02	49.08 ± 9.42**	48.92 ± 8.98**

Data are presented as mean ± SD (normal weight, *n* = 14; obesity, *n* = 13). BMI-group-by-time interaction *p* values were: weight < 0.001; BMI < 0.001; waist-to-hip ratio = 0.662; BIA-derived fat mass = 0.080; body-fat percentage = 0.233; subcutaneous fat = 0.105; visceral-fat level = 0.049; and muscle mass = 0.067. Asterisks denote Holm-adjusted within-group contrasts versus baseline (*p < 0.05, **p < 0.01) and do not establish a between-group difference in response. InBody 770 body-composition outputs are hydration-sensitive estimates and should not be interpreted as directly measured changes in adipose or muscle tissue.

Prespecified exploratory sex-stratified summaries are retained in Supplementary Table S1. Among normal-weight women (*n* = 8), nominal pre-to-post-fasting changes were observed for waist-to-hip ratio and the BIA-derived fat-mass, muscle-mass, and subcutaneous-fat estimates; among normal-weight men (*n* = 6), body weight, BMI, and the muscle-mass estimate changed nominally. Within the obesity stratum, men (*n* = 8) showed nominal changes in body weight, BMI, and most BIA-derived estimates, whereas the five women with complete body-composition data showed changes in the same general direction for several measures but no nominal *p* value below 0.05. These are separate within-subgroup descriptions based on uncorrected *p* values, not evidence that responses differed by sex.

### 3.3 Changes in BIA-derived phase angle

PhA was analyzed as an impedance-derived indicator related to cellular membrane properties and hydration status (Table 2). The two-factor repeated-measures model showed a significant overall time effect, *F*_(1.88, 46.93)_ = 22.05, *p* < 0.001, *η_p_*^2^ = 0.469, but no BMI-group-by-time interaction, *F*_(1.88, 46.93)_ = 0.13, *p* = 0.867, *η_p_*^2^ = 0.005. In the normal-weight group, PhA changed from 5.63 ± 0.48° at baseline to 5.35 ± 0.54° at 1 w and 5.89 ± 0.78° at 2 w. In the obesity group, the corresponding values were 4.94 ± 0.54°, 4.74 ± 0.57°, and 5.23 ± 0.52°. Holm-adjusted contrasts indicated that the 1 w-to-2 w increase was significant in both groups, whereas the baseline-to-1 w and baseline-to-2 w contrasts were not consistently significant across groups.

**Table 2:** BIA-derived phase angle during fasting and refeeding.

Indicator	Normal weight group (*n* = 14)			Obesity group (*n* = 13)		
	Pre-fasting (0 w)	Post-fasting (1 w)	Refeeding (2 w)	Pre-fasting (0 w)	Post-fasting (1 w)	Refeeding (2 w)
Phase angle (°)	5.63 ± 0.48	5.35 ± 0.54*	5.89 ± 0.78^*##^	4.94 ± 0.54	4.74 ± 0.57	5.23 ± 0.52^##^

Data are presented as mean ± SD (normal weight, *n* = 14; obesity, *n* = 13). The BMI-group-by-time interaction was not significant (*p* = 0.867). Asterisks denote Holm-adjusted contrasts versus baseline (*p < 0.05, **p < 0.01); pound signs denote Holm-adjusted contrasts versus 1 w (#*p* < 0.05, ##*p* < 0.01).

The observed PhA trajectory is compatible with altered fluid distribution during fasting and refeeding. However, PhA is itself impedance-derived and cannot determine how much of the accompanying change reflects water, glycogen, adipose tissue, or muscle tissue. Its rebound after refeeding supports cautious interpretation of the InBody outputs but does not prove the absence of adipose- or muscle-tissue change or exclude proteolysis.

The sex-stratified PhA summaries in Supplementary Table S1 provide additional descriptive detail. Normal-weight men showed a nominal decline from 6.02 ± 0.38° to 5.62 ± 0.19° at 1 w (*p* = 0.045) and a higher value at 2 w (6.55 ± 0.30°, *p* = 0.004 versus baseline), whereas normal-weight women showed no nominal change. In the obesity stratum, women showed a nominal decline at 1 w (4.70 ± 0.21° to 4.36 ± 0.28°, *p* = 0.018) followed by return toward baseline, while men showed no nominal 1 w change but a higher 2 w value (5.49 ± 0.46°, *p* = 0.033 versus baseline). No sex-by-time interaction was tested, so these patterns should not be interpreted as evidence of different trajectories by sex.

### 3.4 Changes in upper- and lower-limb strength-test performance

The two-factor repeated-measures model showed significant overall time effects for grip, knee-flexion, and knee-extension dynamometer scores (all Greenhouse–Geisser-adjusted *p* < 0.001). In the normal-weight group, the knee-extension and knee-flexion scores were 6.84 kg and 2.37 kg higher immediately after fasting, while the grip score was 3.06 kg higher. At 2 w, the recorded grip, knee-extension, and knee-flexion scores were 3.80, 3.96, and 3.44 kg above baseline, respectively.

In the obesity group, the recorded grip, knee-flexion, and knee-extension scores were 4.65, 2.98, and 8.78 kg higher immediately after fasting. At 2 w, the corresponding scores remained 4.77, 3.51, and 4.65 kg above baseline.

BMI-group-by-time interactions were not significant for grip, *F*_(1.99, 49.70)_ = 0.37, *p* = 0.691, *η_p_*^2^ = 0.015; knee flexion, *F*_(1.40, 35.06)_ = 0.18, *p* = 0.753, *η_p_*^2^ = 0.007; or knee extension, *F*_(1.24, 31.01)_ = 1.01, *p* = 0.340, *η_p_*^2^ = 0.039. Thus, the data do not show different temporal trajectories between BMI groups. The higher later scores document maintained or increased measured test performance, but the absence of a non-fasting control and a dedicated familiarization protocol prevents separation of biological adaptation from learning or measurement effects (Table 3).

**Table 3:** Upper- and lower-limb dynamometer test performance during fasting and refeeding.

Indicator	Normal weight group (*n* = 14)			Obesity group (*n* = 13)		
	Pre-fasting (0 w)	Post-fasting (1 w)	Refeeding (2 w)	Pre-fasting (0 w)	Post-fasting (1 w)	Refeeding (2 w)
Grip dynamometer score (kg)	30.91 ± 13.57	33.97 ± 12.76	34.71 ± 13.16^*^	36.68 ± 11.17	41.33 ± 12.51^**^	41.45 ± 12.86^*^
Knee-flexion dynamometer score (kg)	10.65 ± 3.00	13.02 ± 3.75^**^	14.09 ± 3.95**	13.37 ± 3.33	16.35 ± 4.45^*^	16.88 ± 4.99^*^
Knee-extension dynamometer score (kg)	13.00 ± 3.99	19.84 ± 5.03**	16.96 ± 4.35**	16.37 ± 4.59	25.15 ± 4.63^**^	21.01 ± 4.28^**^

Data are presented as mean ± SD (normal weight, *n* = 14; obesity, *n* = 13). BMI-group-by-time interaction *p* values were 0.691 for grip, 0.753 for knee flexion, and 0.340 for knee extension. Asterisks denote Holm-adjusted within-group contrasts versus baseline (*p < 0.05, **p < 0.01) and do not establish a between-group difference in response. Values are dynamometer-recorded test scores and may be influenced by familiarization or measurement effects.

The exploratory sex-stratified dynamometer summaries are reported in Supplementary Table S2. Normal-weight women showed nominal increases in knee-extension scores after fasting and in knee-flexion and knee-extension scores after refeeding; normal-weight men showed nominal increases in knee-flexion scores and in the knee-extension score immediately after fasting. Among obesity-group participants with complete dynamometry data, men showed nominal increases across all three tests at 1 w and in both knee tests at 2 w, whereas women showed numerical increases but no nominal *p* value below 0.05. These within-subgroup patterns do not establish sex-specific physiological gains and remain susceptible to familiarization and measurement effects.

### 3.5 Changes in blood pressure, heart rate, and vascular-stiffness indicators

The two-factor repeated-measures model showed significant overall time effects for heart rate, *F*_(1.43, 35.63)_ = 18.77, *p* < 0.001, *η_p_*^2^ = 0.429; augmentation index, *F*_(1.88, 46.94)_ = 6.34, *p* = 0.004, *η_p_*^2^ = 0.202; and pulse-wave velocity, *F*_(1.84, 45.88)_ = 4.59, *p* = 0.018, *η_p_*^2^ = 0.155. The time effects for diastolic and systolic blood pressure were not significant (*p* = 0.195 and *p* = 0.098, respectively). Heart rate rose at 1 w and returned toward baseline at 2 w in both BMI groups; other vascular changes were smaller and should be considered exploratory.

None of the cardiovascular outcomes showed a significant BMI-group-by-time interaction: diastolic blood pressure, *p* = 0.897; systolic blood pressure, *p* = 0.756; heart rate, *p* = 0.367; augmentation index, *p* = 0.626; and pulse-wave velocity, *p* = 0.541. Therefore, the data do not support different cardiovascular response trajectories between the BMI groups. Without a non-fasting control, the observed temporal changes cannot be attributed specifically to fasting (Table 4).

**Table 4:** Blood pressure, heart rate, and vascular-stiffness indicators during fasting and refeeding.

Indicator	Normal weight group (*n* = 14)			Obesity group (*n* = 13)		
	Pre-fasting (0 w)	Post-fasting (1 w)	Refeeding (2 w)	Pre-fasting (0 w)	Post-fasting (1 w)	Refeeding (2 w)
DBP (mmHg)	73.86 ± 11.20	74.71 ± 10.89	72.07 ± 13.96	83.92 ± 11.25	84.08 ± 8.09	80.62 ± 10.87
SBP (mmHg)	111.14 ± 12.84	108.57 ± 11.53	107.50 ± 14.87	119.38 ± 10.12	118.38 ± 10.24	114.77 ± 9.78
Heart rate (bpm)	74.14 ± 8.08	89.71 ± 15.45**	72.93 ± 9.15	73.31 ± 10.84	85.31 ± 11.83^*^	75.62 ± 12.70
AIX	24.32 ± 9.10	29.24 ± 13.75	16.96 ± 9.83	20.85 ± 11.84	23.85 ± 9.10	16.77 ± 13.28
PWV (m/s)	6.06 ± 0.97	5.94 ± 0.93	5.90 ± 0.97	7.01 ± 1.20	6.96 ± 1.26	6.79 ± 1.32

Data are presented as mean ± SD (normal weight, *n* = 14; obesity, *n* = 13). BMI-group-by-time interaction *p* values were 0.897 for DBP, 0.756 for SBP, 0.367 for heart rate, 0.626 for augmentation index, and 0.541 for pulse-wave velocity. Asterisks denote Holm-adjusted within-group contrasts versus baseline (*p < 0.05, **p < 0.01) and do not establish a between-group difference in response.

Supplementary Table S3 retains the exploratory sex-stratified cardiovascular summaries. At 1 w, normal-weight women showed nominal changes in heart rate and augmentation index, while normal-weight men showed nominal reductions in systolic blood pressure and pulse-wave velocity. In the obesity stratum, men showed a nominal heart-rate increase at 1 w and a lower pulse-wave velocity at 2 w; the five women with complete cardiovascular data showed no nominal change. Because these are separate within-subgroup tests and no sex-by-time interaction was estimated, the results describe subgroup patterns without demonstrating differential cardiovascular responses by sex.

### 3.6 Changes in gut microbiome alpha diversity before and after short-term intensive fasting

Alpha diversity was largely resilient to the fast (Table 5). In the normal-weight group, the ACE index declined significantly at 1 w (*p* = 0.005), indicating reduced community richness, and this reduction survived Benjamini–Hochberg FDR correction (*q* = 0.021); Chao1, Shannon, and Simpson indices were unchanged. In the obese group, none of the four indices changed significantly.

**Table 5:** Changes in gut microbiome alpha diversity before and after short-term intensive fasting.

Diversity index	Normal weight group (*n* = 14)		Obese group (*n* = 14)	
	Pre-fasting (0 w)	Post-fasting (1 w)	Pre-fasting (0 w)	Post-fasting (1 w)
Chao index	185.37 ± 60.16	165.16 ± 44.83	200.08 ± 47.65	180.23 ± 55.36
Ace index	181.67 ± 52.75	160.64 ± 42.76**	194.51 ± 48.05	179.76 ± 53.91
Shannon index	3.06 ± 0.56	2.95 ± 0.47	3.24 ± 0.29	3.20 ± 0.49
Simpson index	0.12 ± 0.08	0.13 ± 0.07	0.09 ± 0.03	0.10 ± 0.06

Data are presented as mean ± SD (normal-weight: *n* = 14; obese: *n* = 14). Within-group comparisons (0w vs. 1w) by Wilcoxon signed-rank test with Benjamini-Hochberg FDR correction across the four indices. Bold values indicate significance after FDR correction (*q* < 0.05). *p < 0.05 (nominal), **p < 0.01 (nominal).

Supplementary Table S4 presents the sex-stratified alpha-diversity results. None of the Chao1, ACE, Shannon, or Simpson comparisons reached nominal *p* < 0.05 in any sex-by-BMI subgroup; the largest numerical signal was the Chao1 decline in obesity-group men (*p* = 0.058). These small-subgroup results are compatible with relative stability of alpha diversity but cannot establish that sex had no influence.

### 3.7 Compositional changes at the phylum and genus levels

**Figure 2:**
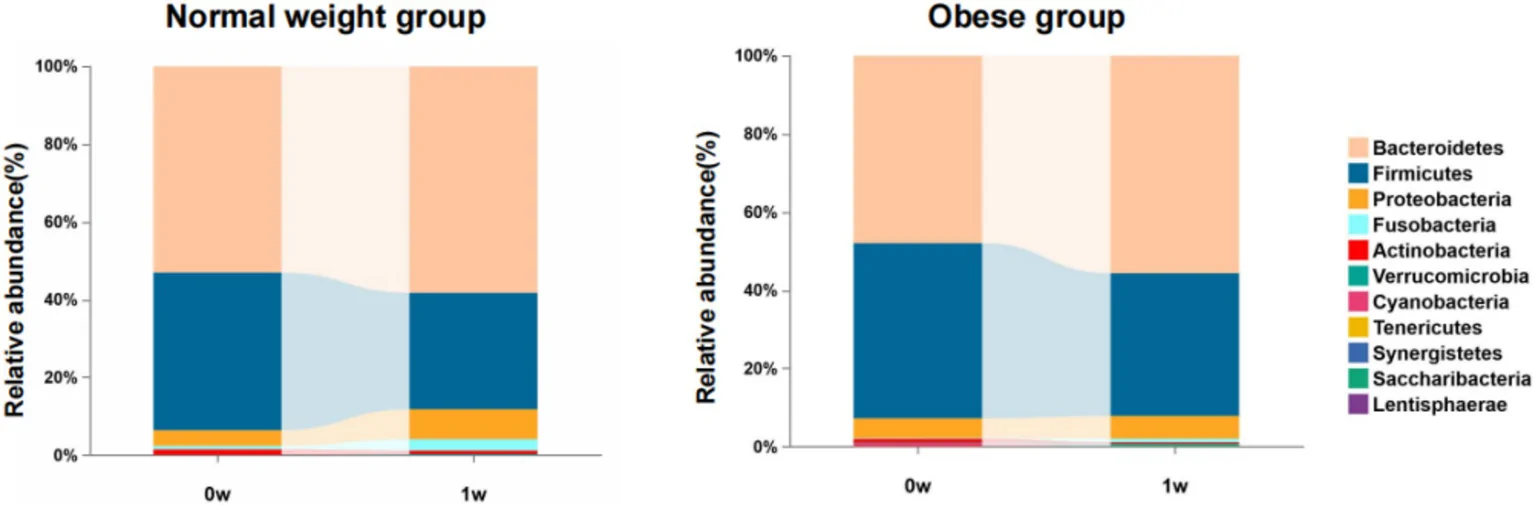
Changes in gut microbiota composition at the phylum level before and after short-term intensive fasting. Abundance values are in percentage (%); data are presented as mean (normal-weight: *n* = 14; obese: *n* = 14).

**Figure 3:**
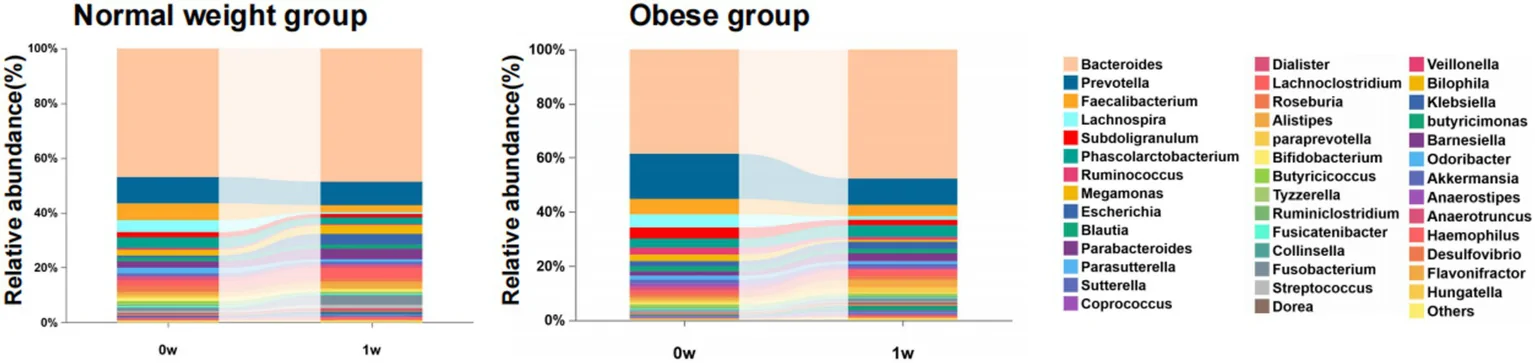
Changes in gut microbiota composition at the genus level before and after short-term intensive fasting. Abundance values are in percentage (%); Data are presented as mean (normal-weight: *n* = 14; obese: *n* = 14).

At the phylum and genus levels, 7 days of fasting remodeled gut microbiota composition in both groups, although most taxonomic shifts did not survive Benjamini–Hochberg FDR correction (Tables 6, 7; Figures 2, 3).

**Table 6:** The impact of short-term intensive fasting on the phylum level of gut microbiota.

Phylum	Pre-fasting	Post-fasting	*p* (nominal)	*q* (FDR)
Normal weight group				
*Firmicutes*	40.51 ± 8.04	29.95 ± 11.33*	0.0134	0.1943
*Proteobacteria*	4.02 ± 3.77	7.70 ± 5.37*	0.0295	0.2671
*Fusobacteriia*	0.93 ± 1.87	2.93 ± 5.03**	0.0071	0.1407
Obese group				
*Firmicutes*	44.78 ± 8.38	36.47 ± 14.75*	0.0245	0.1656
*Actinobacteria*	0.90 ± 0.68	0.39 ± 0.49**	0.0012	0.0824

All taxa with nominal *p* < 0.05 are listed. Abundance values are in percentage (%); Data are presented as mean ± SD (normal-weight: *n* = 14; obese: *n* = 14). Within-group comparisons by Wilcoxon signed-rank test; *p*-values were then subjected to Benjamini-Hochberg FDR correction within each comparison family. Given the exploratory nature of this pilot study, taxa with nominal *p* < 0.05 but FDR *q* ≥ 0.05 are retained as exploratory observations warranting validation in larger cohorts.

**Table 7:** Changes in the abundance of gut microbiota at the genus level.

Genus	Pre-fasting	Post-fasting	*p* (nominal)	*q* (FDR)
Normal weight group				
*Faecalibacterium*	5.15 ± 3.82	2.26 ± 2.45*	0.0494	0.3285
*Phascolarctobacterium*	3.27 ± 2.23	2.10 ± 1.65*	0.0392	0.3041
*Lachnospira*	3.68 ± 3.00	0.57 ± 1.39**	0.0067	0.1407
*Parabacteroides*	1.98 ± 1.72	3.42 ± 3.07*	0.0295	0.2671
*Lachnoclostridium*	1.73 ± 1.71	3.40 ± 3.30*	0.0353	0.2944
*Fusobacterium*	0.93 ± 1.87	2.93 ± 5.03**	0.0071	0.1407
*Coprococcus*	0.28 ± 0.56	0.21 ± 0.50*	0.0166	0.2001
*Hungatella*	0.021 ± 0.025	0.26 ± 0.32**	0.0067	0.1407
Obese group				
*Bacteroides*	30.58 ± 15.65	38.92 ± 18.41*	0.0134	0.1261
*Lachnospira*	3.83 ± 2.59	1.10 ± 1.67**	0.0023	0.0824
*Parabacteroides*	1.25 ± 1.19	2.39 ± 1.78*	0.0245	0.1656
*Subdoligranulum*	3.23 ± 2.99	1.63 ± 2.19*	0.0245	0.1656
*Megamonas*	1.91 ± 4.40	0.61 ± 1.60*	0.0117	0.1261
*Ruminococcus*	2.06 ± 1.72	0.85 ± 1.17**	0.0031	0.0824
*Butyricimonas*	0.18 ± 0.35	1.17 ± 2.99*	0.0342	0.2171
*Hungatella*	0.021 ± 0.034	0.16 ± 0.28*	0.0166	0.1379

All taxa with nominal *p* < 0.05 are listed. Abundance values are in percentage (%); Data are presented as mean ± SD (normal-weight: *n* = 14; obese: *n* = 14). Within-group comparisons by Wilcoxon signed-rank test; *p*-values were then subjected to Benjamini-Hochberg FDR correction within each comparison family. Given the exploratory nature of this pilot study, taxa with nominal *p* < 0.05 but FDR *q* ≥ 0.05 are retained as exploratory observations warranting validation in larger cohorts.

At the phylum level, the normal-weight group showed a decrease in *Firmicutes* (nominal *p* = 0.013) alongside increases in *Proteobacteria* (*p* = 0.030) and *Fusobacteria* (*p* = 0.007), whereas the obese group exhibited reductions in *Firmicutes* (*p* = 0.026) and *Actinobacteria* (*p* = 0.003). Only the enrichment of *Pseudomonadales* (order level) in the normal-weight group survived FDR correction (*q* = 0.027); all other phylum-level changes should be considered exploratory.

At the genus level, the normal-weight group showed reductions in several butyrate-producing genera—*Faecalibacterium*, *Lachnospira*, and *Coprococcus*—together with increases in *Parabacteroides* and *Fusobacterium*. In the obese group, fasting enriched *Bacteroides* and *Parabacteroides* while depleting *Ruminococcus*, *Megamonas*, and *Subdoligranulum*. These shifts point to directional remodeling toward *Bacteroides*-associated taxa and away from pro-inflammatory genera, although confirmation in larger cohorts is warranted.

Compositional changes at the class (Supplementary Table S6), order (Supplementary Table S7), family (Supplementary Table S8), and species (Supplementary Table S9) levels are provided in Supplementary material and were generally consistent with the phylum- and genus-level patterns described above.

### 3.8 Differences in physical health indicators between the two groups

**Figure 4:**
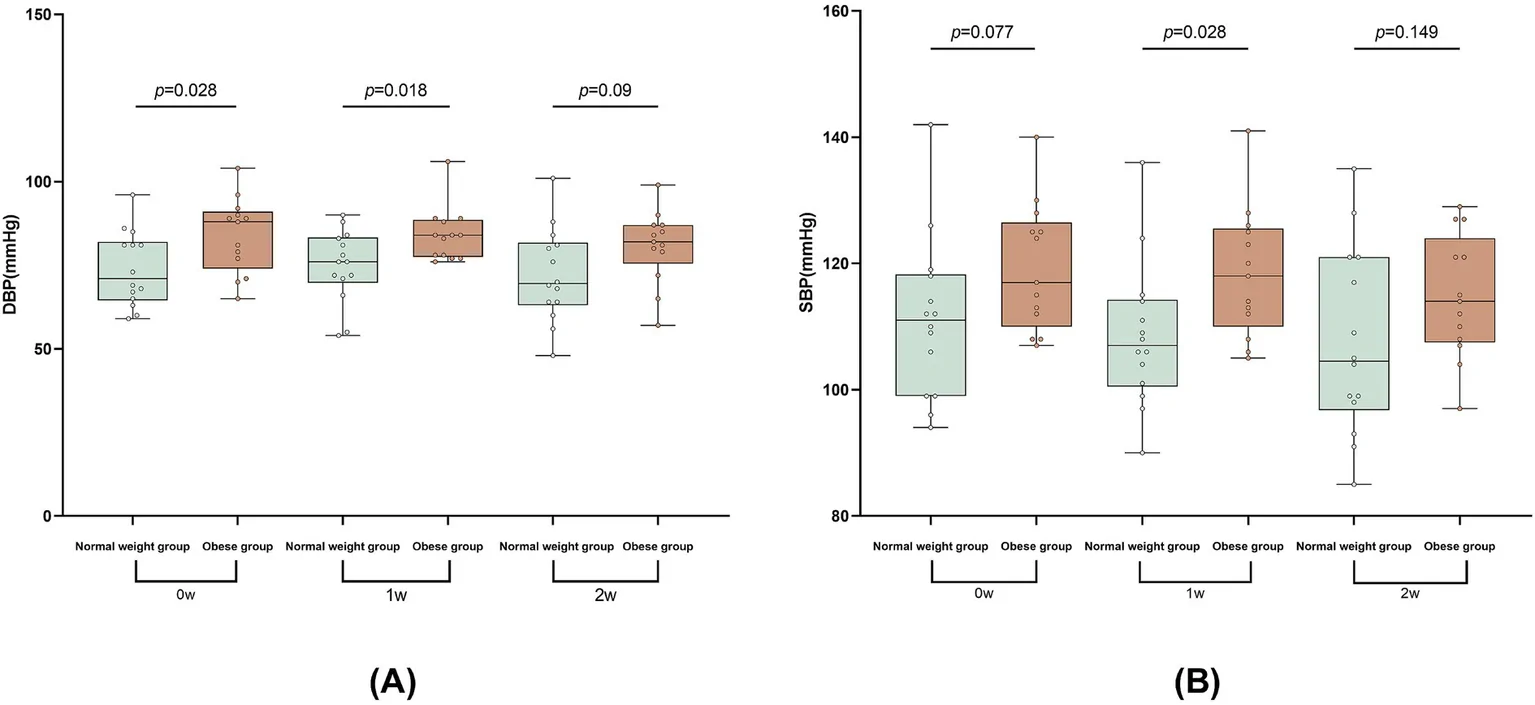
Blood pressure changes in obese vs. normal-weight groups during and post-fasting intervention **(A)** DBP across three phases: Pre-fasting (0 w), post-fasting (1 w), and post-refeeding (2 w); **(B)** SBP comparisons under the same timeline. Data are presented as mean ± SD (normal-weight: *n* = 14; obese: *n* = 13). Error bars represent SD. Between-group comparisons at each time point by Mann–Whitney U test (nominal *p* values, exploratory).

Exploratory between-group comparisons at individual time points are shown in Figure 4. In the primary repeated-measures models, however, the BMI-group-by-time interactions were not significant for diastolic pressure, systolic pressure, or pulse-wave velocity (all *p* > 0.54). Consequently, changes in whether a between-group comparison crossed *p* = 0.05 at separate time points were not interpreted as evidence that the groups converged or responded differently.

### 3.9 Changes in the differences in gut microbiota composition between the two groups

**Figure 5:**
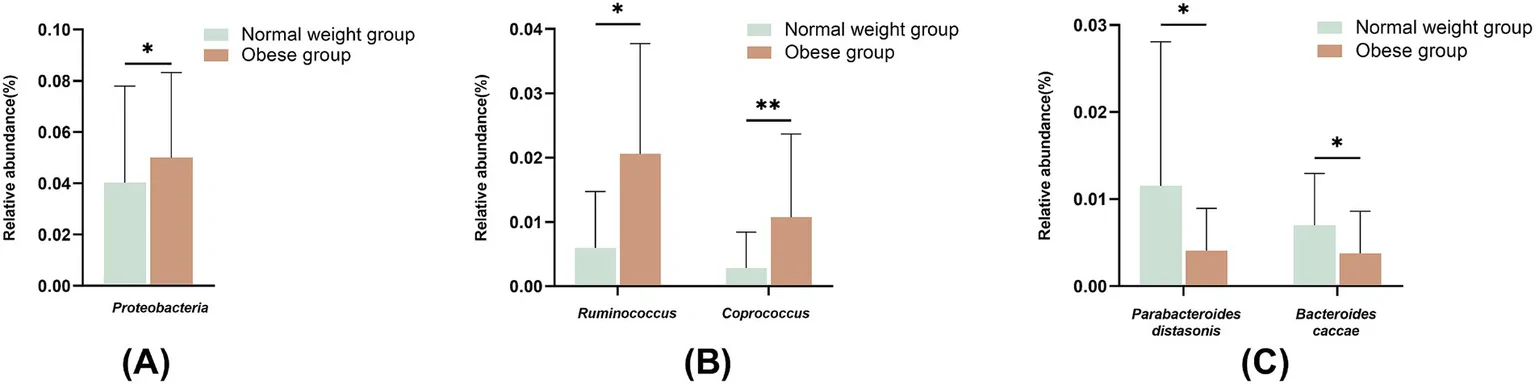
Between-group differences in gut microbiota composition before and after short-term intensive fasting. **(A)** Relative abundance of *Proteobacteria* at the phylum level; **(B)** Genus-level comparison of *Ruminococcus* and *Coprococcus*; **(C)** Species-specific differences in *Parabacteroides distasonis* and *Bacteroides caccae*. Open bars = pre-fasting (0 w); solid bars = post-fasting (1 w). Values represent mean relative abundance (%) ± SD (normal-weight: *n* = 14; obese: *n* = 14). Error bars represent SD. Between-group comparisons by Mann–Whitney U test (nominal *p* values; none survived FDR correction). * *p* < 0.05, ** *p* < 0.01 (nominal).

Before fasting, the normal-weight group showed nominally lower abundances of *Proteobacteria* (*Z* = −2.022, *p* = 0.043), *Ruminococcus* (*Z* = −2.574, *p* = 0.010), and *Coprococcus* (*Z* = −3.216, *p* = 0.001), and nominally higher abundances of *Parabacteroides distasonis* (*Z* = −2.205, *p* = 0.027) and *Bacteroides caccae* (*Z* = −2.389, *p* = 0.017) than the obese group (Table 8). After 7 days of fasting, none of these between-group contrasts remained significant (*Proteobacteria*: *p* = 0.183; *Ruminococcus*: *p* = 0.287; *Coprococcus*: *p* = 0.054; *Parabacteroides distasonis*: *p* = 0.335; *Bacteroides caccae*: *p* = 0.462) (Figure 5).

**Table 8:** Differences in gut microbiota composition between normal-weight and obese groups (%).

Bacterial name	Normal weight group		Obese group	
	0 w	1 w	0 w	1 w
*Proteobacteria*	4.02 ± 3.77	7.70 ± 5.37	4.99 ± 3.33*	5.84 ± 4.45
*Ruminococcus*	0.60 ± 0.88	0.38 ± 0.94	2.06 ± 1.72*	0.85 ± 1.17
*Coprococcus*	0.28 ± 0.56	0.21 ± 0.50	1.07 ± 1.30**	0.61 ± 1.08
*Parabacteroides_distasonis*	1.15 ± 1.66	1.85 ± 2.84	0.41 ± 0.49*	0.92 ± 0.93
*Bacteroides_caccae*	0.70 ± 0.60	0.77 ± 1.08	0.38 ± 0.48*	1.32 ± 1.83

All taxa with nominal *p* < 0.05 are listed. Data are presented as mean ± SD (normal-weight: *n* = 14; obese: *n* = 14). Between-group comparisons by Mann–Whitney U test; *p*-values were then subjected to Benjamini-Hochberg FDR correction within each comparison family. Given the exploratory nature of this pilot study, taxa with nominal *p* < 0.05 but FDR *q* ≥ 0.05 are retained as exploratory observations warranting validation in larger cohorts.

Two caveats temper this apparent convergence. First, none of the baseline between-group differences survived Benjamini–Hochberg FDR correction (all *q* ≥ 0.05), so the baseline divergence itself is exploratory. Second, PERMANOVA detected no significant difference in overall community composition between groups either at baseline (*p* = 0.143) or post-fasting (*p* = 0.348; see Section 3.10). The disappearance of nominal taxon-level differences therefore reflects directional shifts in specific taxa rather than a statistically robust convergence of community structure, and these observations require validation in larger cohorts (Supplementary Table S10).

### 3.10 Beta diversity analysis of gut microbiota

**Figure 6:**
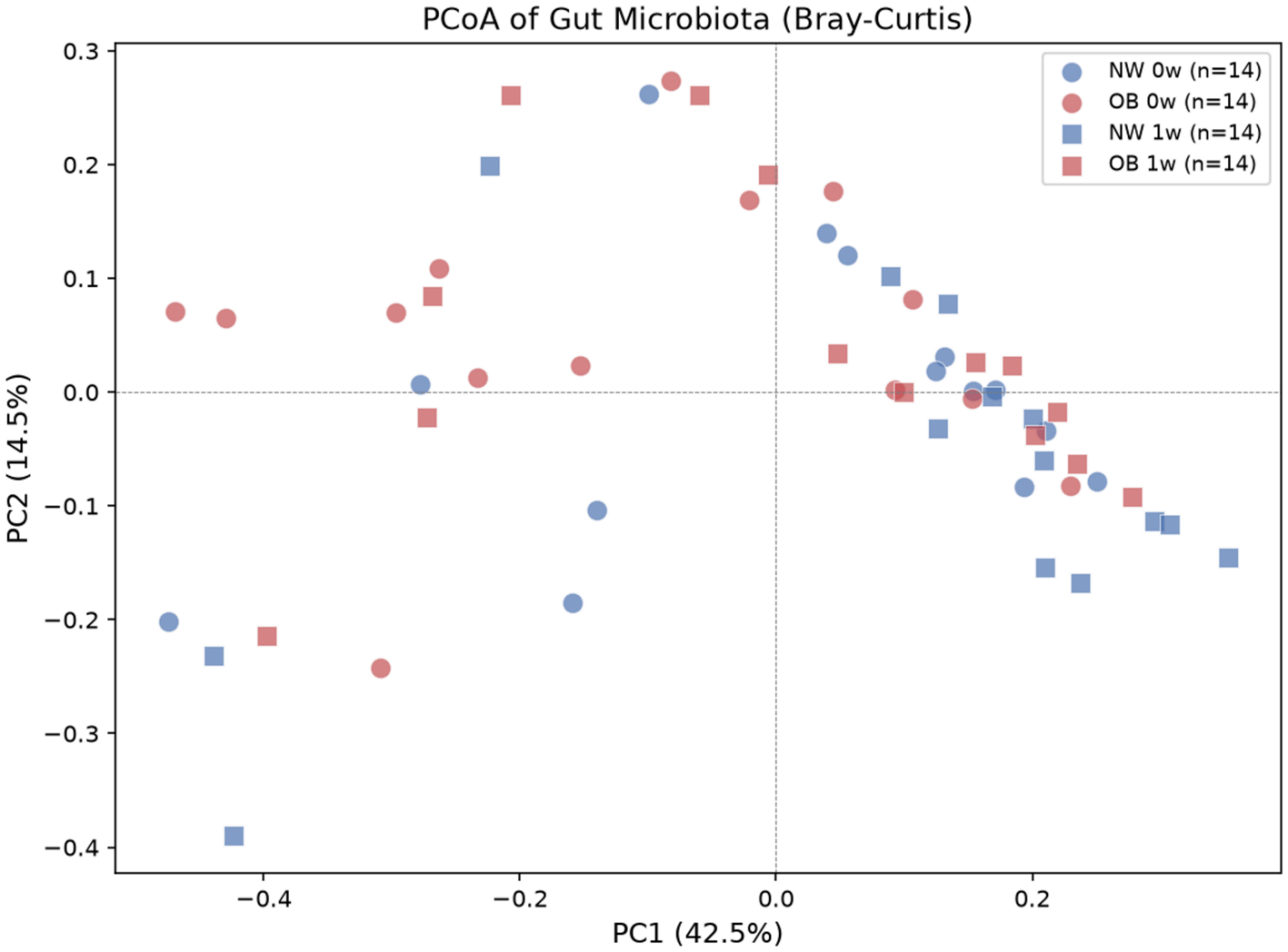
Principal coordinates analysis (PCoA) of genus-level gut microbiota composition based on Bray–Curtis dissimilarity. Each point represents one sample (*n* = 56: 14 normal-weight + 14 obese participants × 2 time points). NW, normal-weight; OB, obese. Open circles = pre-fasting (0 w); solid squares = post-fasting (1 w). Percentages indicate variance explained by each principal coordinate. PERMANOVA (999 permutations): 0 w Group comparison *p* = 0.143; 1 w Group comparison *p* = 0.348.

To test directly whether the gut communities of obese and normal-weight participants converged during fasting, we performed β-diversity analysis using Bray–Curtis dissimilarity and PCoA at the genus level (Figure 6). PERMANOVA detected no significant between-group difference at baseline (*F* = 29.50, *p* = 0.143) or after the fast (*F* = 28.17, *p* = 0.348), and the four-group comparison (NW_0w, NW_1w, OB_0w, OB_1w) likewise fell short of significance (*F* = 20.84, *p* = 0.063). The within-subject Bray–Curtis distance between paired 0 w and 1 w samples—indexing the magnitude of fasting-induced remodeling—was comparable between normal-weight (0.347 ± 0.092) and obese (0.306 ± 0.125) participants (Mann–Whitney *U* test, *p* = 0.346). Consistent with this, the PCoA ordination (PC1: 42.5%; PC2: 14.5% of variance) showed substantial overlap among the four groups, with no clear separation by group or time point.

Thus, at the community level, the gut microbiota of the two groups differed significantly neither before nor after fasting, and the taxon-level shifts described above represent changes in the relative abundance of specific taxa rather than wholesale convergence of community structure.

### 3.11 Safety and tolerability

The 7-day water-only fast was well tolerated under medical supervision: no serious adverse events, clinically significant hypoglycemia, electrolyte crises, or refeeding syndrome occurred in either group. Expected mild, transient symptoms arose during the first 48–72 h of fasting—fatigue in 35.7% (*n* = 10) of participants, headache in 21.4% (*n* = 6), and orthostatic dizziness in 17.9% (*n* = 5)—and all resolved spontaneously or during early refeeding without intervention.

## 4 Discussion

This pilot study examined short-term physiological and gut-microbiota changes during a supervised seven-day water-only fast followed by structured refeeding in adults with obesity and normal-weight controls. Directly measured body weight showed the clearest change, with a larger trajectory in the obesity group. BIA-derived estimates, dynamometer-recorded test performance, cardiovascular indicators, and microbiome profiles provided complementary signals, but each requires interpretation according to its measurement and multiplicity limitations. The resulting pattern is informative for hypothesis generation while remaining distinct from confirmatory evidence of tissue change, physiological strength enhancement, or causal microbiome remodeling.

### 4.1 Short-term intensive fasting and physical health indicators

#### 4.1.1 Interpretation of body weight and BIA-derived estimates

Body weight declined during the seven-day water-only fast. The composition of that weight change, however, cannot be established from the InBody 770 outputs. Bioelectrical impedance estimates body water and fat-free mass from electrical measurements and prediction algorithms, with fat mass derived indirectly. Consensus guidance cautions that BIA results are unreliable or require particular caution when hydration is abnormal (33), and acute hydration changes can materially alter multifrequency BIA estimates (34). Accordingly, the 3.28-kg decrease in estimated fat mass in the obesity group is reported as a change in a BIA-derived value under hydration-perturbed conditions, not as measured adipose-tissue loss.

The simultaneous decreases in BIA-derived fat and muscle estimates are both vulnerable to fasting-related changes in total-body-water distribution, glycogen-associated water, and electrolytes. The U-shaped PhA trajectory is compatible with reversible fluid redistribution, but neither PhA nor the proprietary body-composition outputs can quantify true tissue change. Because no criterion method such as DXA, MRI, or isotope-dilution assessment was performed, the present data cannot partition the observed weight loss into adipose tissue, lean tissue, glycogen, and water.

Body weight and several BIA-derived estimates remained below baseline after refeeding. Persistence of an impedance-derived estimate does not remove the hydration limitation or confirm tissue loss, particularly because refeeding can continue to alter glycogen and fluid distribution. Future studies should combine standardized hydration assessment with a criterion body-composition method and longer follow-up.

The formal BMI-group-by-time interactions indicated different trajectories for body weight and BMI, with larger absolute decreases in the obesity group. In contrast, the interactions for BIA-derived fat mass, body-fat percentage, subcutaneous fat, and muscle mass were not significant, and the visceral-fat-level interaction was only borderline. Thus, the data support a between-group difference in measured weight and BMI trajectories, but not a confident claim that adipose- or muscle-tissue responses differed.

#### 4.1.2 Interpretation of strength-test performance, blood pressure, heart rate, and vascular stiffness

Dynamometer-based tests are commonly used to characterize physical performance. Higher grip, knee-flexion, and knee-extension scores were recorded at later assessments, including approximately 6.84-kg and 8.78-kg increases in knee-extension scores immediately after fasting in the normal-weight and obesity groups. The consistent direction across several tests indicates preserved or higher measured performance during the study period, but the magnitude of the knee-extension changes warrants a cautious interpretation.

Both biological maintenance and test-related explanations remain possible. Repeated dynamometer testing can produce familiarization and motor-learning effects, while motivation, positioning, stabilization, instruction, and device or operator variability can influence recorded force. Because no non-fasting group underwent the same testing schedule, the present design cannot quantify the contribution of fasting relative to these alternative explanations. The findings therefore support a conclusion of maintained or increased measured test performance, not confirmed enhancement of physiological muscular strength.

Future studies should include a dedicated familiarization session, standardized repeated trials with prespecified averaging rules, assessor blinding where feasible, test–retest reliability assessment, and a contemporaneous control group. Such designs could determine whether any component of the observed performance trajectory exceeds expected practice-related variation.

Beyond musculoskeletal function, the cardiovascular measurements provided short-term safety information. Heart rate rose after fasting and returned toward baseline after refeeding, a pattern compatible with adaptation to sustained energy deprivation and sympathetic activation (43). The two participants with elevated pre-fasting diastolic pressure (92 and 104 mmHg) measured 83 and 106 mmHg after fasting and 72 and 99 mmHg after refeeding, respectively; these individual observations are descriptive rather than evidence of an antihypertensive effect. No serious adverse event or event affecting daily functioning was recorded, supporting the acute tolerability of the supervised protocol in this cohort.

Arterial stiffness, indexed by AIX and PWV (35–37), is associated with cardiovascular and cerebrovascular risk (44), and supervised water-only fasting has previously been linked to reduced thrombotic risk (24). AIX in the normal-weight group and PWV in the obesity group were numerically lower after refeeding (normal-weight AIX: *p* = 0.122; obesity-group PWV: *p* = 0.074), but the BMI-group-by-time interactions were not significant and the within-group trends did not provide confirmatory evidence of vascular improvement. Together with the observed weight and BMI trajectories, these signals justify longer, adequately powered studies with standardized vascular endpoints rather than a claim of cardiovascular efficacy.

### 4.2 Short-term intensive fasting and the gut microbiota

The gut microbiota regulates host metabolism through nutrient assimilation, vitamin synthesis (e.g., K2, B12), and xenobiotic detoxification, placing it at the interface of diet and obesity pathogenesis (45). Dietary interventions reshape it rapidly: a 12-week IF trial increased *Bacteroidetes* and lowered the *Firmicutes*/*Bacteroidetes* ratio in parallel with improved insulin sensitivity (46); 8-h time-restricted feeding enriched butyrate-producing *Lachnospiraceae* while suppressing LPS-generating *Enterobacteriaceae*, attenuating systemic inflammation (47); and caloric restriction enriched *Akkermansia muciniphila*, a mucin degrader that strengthens barrier function, and the heritable anti-obesity taxon *Christensenellaceae* (48). The nominal compositional shifts we observed point in the same direction—in the obese group, enrichment of *Bacteroides* (*p* = 0.013) and reduction of *Ruminococcus* (*p* = 0.003)—although none of these genus-level changes survived Benjamini–Hochberg FDR correction across the 868 taxonomic comparisons examined (all *q* ≥ 0.05); only *Pseudomonadales* (order level, normal-weight group) remained significant (*q* = 0.027). These observations are therefore exploratory and hypothesis-generating rather than evidence of microbiota normalization.

Mechanistically, SCFAs such as propionate and butyrate can activate vagal afferent neurons via free fatty acid receptor 3 (FFAR3/GPR41), and this gut–brain signaling modulates hypothalamic appetite centers—suppressing orexigenic neuropeptide Y and enhancing satiety (49, 50). The nominal enrichment of *Bacteroides*, a genus that includes key SCFA producers, is directionally consistent with this framework, but the absence of FDR significance precludes causal inference.

Inter-individual variability is another consideration: the microbiota is shaped by geography, lifestyle, and above all diet, and high-fat, high-sugar, low-fiber patterns drive dysbiosis (51). Dietary restructuring can shift community composition within roughly 4 days (52), providing a plausible window for fasting-induced change. Yet our β-diversity analysis (PERMANOVA) found no significant community-level difference between groups at baseline or post-fasting—individual taxa moved, but the global community architecture proved largely resilient to this short intervention.

#### 4.2.1 The impact of short-term intensive fasting on gut microbiota alpha diversity

Alpha diversity was essentially unchanged by the fast. In the obese group, none of the Chao1, ACE, Shannon, or Simpson indices shifted significantly; in the normal-weight group, only the ACE index declined, indicating reduced richness (53). A similar stability of diversity has been reported after Ramadan fasting (54), whereas Khan et al. found that time-restricted feeding significantly altered diversity (14), so the field remains divided. The selective ACE reduction is biologically plausible: with luminal carbohydrate supply abruptly cut, substrate-specialist taxa lose their niche and rarity contracts first—a mild ecological bottleneck rather than a collapse of community function (55, 56). Importantly, the ACE reduction was the only microbiome finding in this study to survive FDR correction (*q* = 0.021), making it the most robust microbial signal of this pilot trial.

#### 4.2.2 The impact of short-term intensive fasting on the composition of the gut microbiota

Studies have indicated that calorie restriction affects microbial proliferation, thereby altering the overall state of the gut microbiota. With restricted food intake, the number of microbes that previously thrived on processed foods decreases, which can temporarily lower immune function, but this immune suppression is reversible upon refeeding (17, 57).

As emphasized above, the taxon-specific changes that follow rest on nominal *p* values from Wilcoxon signed-rank tests; after Benjamini–Hochberg correction across all 868 comparisons, only *Pseudomonadales* in the normal-weight group (*q* = 0.027) remained significant, so these patterns are exploratory (55, 58). With that caveat, the two groups showed distinct restructuring trajectories. In normal-weight participants, fasting contracted *Firmicutes*-affiliated taxa—class Clostridia (order *Clostridiales*), families *Ruminococcaceae* (*Faecalibacterium*) and *Lachnospiraceae* (*Lachnoclostridium*), and *Phascolarctobacterium faecium*—while mucolytic and immunomodulatory taxa expanded, including phyla *Proteobacteria* and *Fusobacteria*, genus *Fusobacterium* (var*ium*), and *Bacteroides* (*stercoris*, *ovatus*, *cellulosilyticus*, *nordii*).

In obese participants, fasting preferentially suppressed dysbiosis-linked taxa—phylum *Actinobacteria* (orders *Bifidobacteriales* and *Coriobacteriales*), genera *Ruminococcus* and *Megamonas*, and the pathobionts *Bacteroides plebeius* and *Collinsella aerofaciens*—while barrier-protective taxa were enriched, notably families *Bacteroidaceae* (*Bacteroides*) and *Porphyromonadaceae* (*Parabacteroides*, *Butyricimonas*) and the metabolically beneficial species *Bacteroides thetaiotaomicron* and *Bacteroides caccae*.

Within this exploratory framework, the two BMI strata showed different nominal taxon-level patterns. Several taxa commonly associated with inflammation declined and SCFA-associated genera such as *Bacteroides* and *Parabacteroides* increased, while some potentially beneficial taxa also decreased. These directions are biologically interesting, but their clinical meaning remains uncertain because nearly all taxonomic comparisons failed FDR correction.

*Bacteroidetes* and *Firmicutes* together account for a large proportion of the gut microbiota (59). *Bacteroidetes* are Gram-negative fermenters that yield acetate, propionate, and butyrate and can respond rapidly to dietary change, although individual members may exert different effects (60, 61). After fasting, several Bacteroidetes members increased, whereas several Firmicutes-affiliated taxa declined. This nominal direction resembles patterns reported during caloric restriction and dietary weight loss (9, 62, 63), but it should not be interpreted as evidence that a seven-day fast reproduces the established metabolic effects of longer interventions.

*Proteobacteria* and *Fusobacteria* behave less predictably. *Enterobacteriaceae* often expands during epithelial dysfunction, whereas *Fusobacteria* are opportunists that bloom in malnutrition, cachexia, and diabetes yet can also mount protective inflammatory responses against pathogens (64–66). In our cohort, *Proteobacteria*, *Fusobacteria*, and allied genera increased only in the normal-weight group—plausibly a direct consequence of prolonged nutrient absence, consistent with previous reports (65, 66)—while the obese group showed no significant change. These increases should not be read as unqualified improvements. *Proteobacteria* is a heterogeneous phylum whose expansion marks dysbiosis and low-grade inflammation in some contexts but accompanies weight loss during caloric restriction in others (63, 64); likewise, *Fusobacterium* species are established opportunists implicated in colorectal carcinogenesis and inflammation. Their post-fasting expansion—which, except for *Pseudomonadales*, did not survive FDR correction—is therefore of uncertain biological significance and warrants mechanistic follow-up.

*Lachnospiraceae*, *Ruminococcaceae*, *Lachnoclostridium*, *Roseburia*, and *Eubacterium* have been linked to muscle-mass loss, and *Ruminococcaceae* additionally correlates with type 2 diabetes, NAFLD, and obesity (67). After the fast, *Lachnospiraceae* fell significantly in all participants and *Ruminococcaceae* fell in the obese group—changes that paralleled the observed decline in muscle mass. Conversely, *Bifidobacterium*, *Enterococcus*, and *Streptococcus* ferment fiber into SCFAs (acetate, propionate, butyrate) that stimulate GLP-1 secretion via FFAR2/GPR43 and FFAR3/GPR41 on epithelial and enteroendocrine cells, slowing gastric emptying, delaying hunger, and facilitating weight loss (68). The fall in *Bifidobacterium* in the obese group, with *Enterococcus* and *Streptococcus* unchanged, indicates that fasting also trimmed some beneficial taxa.

Viewed functionally, the compositional shifts formed a coordinated pattern. *Bacteroides*, which can modulate mucosal antiviral immunity (61), and *Clostridium*, a mainstay of intestinal immune homeostasis (69), increased, whereas adverse-outcome taxa declined—*Veillonella*, linked to opportunistic infection and dysbiosis (70), and *Actinomyces*, implicated in inflammatory pathology (71).

Taken together, the nominal findings suggest concurrent increases in several immunomodulatory or barrier-associated taxa and decreases in several inflammation-associated taxa, alongside losses in some beneficial organisms such as Bifidobacterium. The pattern appeared numerically broader in the obesity group and motivates a testable hypothesis that baseline adiposity may influence taxon-level responses to complete fasting; it does not establish amelioration of dysbiosis.

#### 4.2.3 The impact of short-term intensive fasting on the differences in gut microbiota between obese and normal-weight groups

Before fasting, the two groups differed at the taxon level: normal-weight participants carried lower abundances of *Proteobacteria*, *Ruminococcus*, and *Coprococcus* and higher abundances of *Parabacteroides* and *Bacteroides caccae* than obese participants. After the fast, these nominal differences disappeared: *Proteobacteria*, *Parabacteroides*, and *Bacteroides caccae* rose in both groups while *Ruminococcus* and *Coprococcus* fell. Each shift has a plausible physiological reading. *Proteobacteria*, the most functionally variable phylum, is associated with metabolic disorders (72), yet its abundance after caloric restriction correlates positively with weight-loss percentage across ages (73)—consistent with its rise here. *Ruminococcus* correlates positively with obesity and proliferates in inflammatory bowel disease (74); the chronic low-grade inflammation common to both conditions may explain its higher baseline abundance in the obese group. *Coprococcus* expands in high-fat-diet obese mice prone to liver tumorigenesis, where it may partially restrain hepatic inflammation (75). *Parabacteroides*, a commensal inversely correlated with obesity and hepatic steatosis, produces succinate and secondary bile acids that modulate glucose and lipid homeostasis and is considered a promising anti-metabolic-syndrome probiotic (76). *Bacteroides caccae* tends to be more abundant when fasting glucose is low (77, 78), consistent with its increase in both groups here, although its net role—beneficial commensal in healthy adolescents versus enrichment in inflammatory states (79)—remains debated. In sum, the baseline microbiota of normal-weight participants leaned toward health-associated taxa and fewer opportunists; after 7 days of fasting both groups shifted in the same direction, enriching beneficial taxa and trimming potential pathogens, with a partial convergence of taxon-level composition. We nonetheless caution that concurrent components of the intervention—mind–body relaxation in particular—may have influenced autonomic tone and gut transit time and thereby contributed to these microbial dynamics.

Whether the obese microbiota “normalized” toward the normal-weight profile therefore demands a cautious answer. First, the baseline taxon-level differences themselves rested on nominal *p* values and none survived Benjamini–Hochberg correction (all *q* ≥ 0.05), so the starting divergence is exploratory rather than definitive. Second, and more fundamentally, β-diversity analysis (Bray–Curtis, PCoA, PERMANOVA) showed that overall community composition did not differ significantly between groups at baseline or post-fasting, and within-subject compositional change was of similar magnitude in the two groups. The data thus do not support normalization in the strict community-level sense; both groups underwent comparable fasting-induced remodeling without converging on a shared structure.

Accordingly, the taxon-level shifts common to both groups—enrichment of *Bacteroides*-associated species and reduction of *Ruminococcus* and *Coprococcus*—represent directional changes in the relative abundance of specific taxa rather than wholesale convergence of the obese microbiota toward the normal-weight state. Although biologically plausible and consistent with the caloric-restriction literature (63–69), these observations are hypothesis-generating and await validation in larger, independent cohorts.

#### 4.2.4 Exploratory sex-stratified patterns during short-term intensive fasting

The prespecified sex-stratified summaries add descriptive context to the aggregate models. In the obesity stratum, men showed nominal changes across a broader set of weight, BIA-derived, and dynamometer outcomes, whereas women with complete physical-health data showed several changes in the same direction without nominal significance. Normal-weight women and men also showed different configurations of nominal within-subgroup change across BIA-derived estimates, PhA, dynamometer scores, and cardiovascular indicators. These observations identify candidate patterns for future testing but do not demonstrate that sex modified the intervention response.

Several biological factors could plausibly contribute to such numerical heterogeneity, including sex-related differences in fat distribution, resting energy expenditure, skeletal-muscle substrate metabolism, and hormonal regulation of adipose tissue (80–82). However, baseline imbalance, the discrete *p*-value distribution of very small Wilcoxon samples, outcome multiplicity, missing physical-health data in one obesity-group woman, and chance are equally plausible explanations. Accordingly, mechanistic interpretation should remain subordinate to the descriptive nature of the results.

The current sample was not designed for sex-by-time or sex-by-BMI-by-time interaction testing. Future studies should balance recruitment by sex, prespecify interaction hypotheses, standardize hydration assessment, and incorporate dynamometer familiarization and reliability procedures. This approach would preserve the biological question while providing a valid test of whether response trajectories truly differ by sex.

### 4.3 Limitations and prospects

Several limitations qualify these findings. First, the single-arm, pre-post design had no parallel non-fasting or standard caloric-restriction control group; because the intervention bundled behavioral support, mind–body practice, and structured refeeding with complete caloric cessation, the independent effect of energy deprivation per se cannot be isolated, and temporal effects cannot be excluded. The observed physiological and microbial shifts are therefore associations, not proven causal outcomes. Second, the modest pilot sample limits statistical power and generalizability, particularly for high-throughput microbiome profiling and sex-stratified analyses; given the large inter-individual variability imposed by genetics and lifestyle, subtle or low-abundance taxonomic shifts may have escaped detection, inflating false-negative risk. The within-subject paired design, which lets each participant serve as their own control, only partly offsets this constraint. Third, InBody 770 measurements were obtained under fasting-induced changes in hydration; therefore, the observed body-composition changes should be interpreted as BIA-derived estimates rather than confirmed changes in adipose or muscle tissue. Similarly, because dynamometer testing lacked a dedicated familiarization procedure and a non-fasting control, the higher recorded scores may partly reflect learning or measurement effects rather than physiological enhancement of muscular strength. These measurement constraints limit tissue-specific and strength-related interpretation. Fourth, we did not perform comprehensive metabolic phenotyping—insulin-resistance markers, inflammatory profiles, or detailed metabolic biomarkers—so mechanistic interpretation remains incomplete; future studies should incorporate these readouts. Finally, gut microbiota was sampled only before and immediately after the fast, leaving the durability of microbial shifts unknown, and compliance during the home-based phase, though tracked through daily electronic journals and remote check-ins, lacked continuous objective biomarkers such as capillary ketone monitoring or continuous glucose tracking. Future trials should extend post-refeeding follow-up and adopt real-time biomarker verification of adherence.

Notwithstanding these limitations, short-term intensive fasting is inexpensive to implement and showed a favorable acute safety profile, which justifies its further evaluation as an adjunct strategy for metabolic health. Until standardized protocols exist, however, such regimens should be undertaken only under professional supervision.

## 5 Conclusion

In this exploratory pilot study, supervised seven-day water-only fasting followed by structured refeeding was associated with substantial reductions in body weight, with significantly different weight and BMI trajectories between participants with obesity and normal-weight participants. The protocol was also acceptably tolerated under professional supervision. Although several InBody 770 estimates changed during fasting and refeeding, these hydration-sensitive BIA-derived values should not be equated with confirmed changes in adipose or muscle tissue. Dynamometer-recorded test performance was maintained or increased at later assessments; however, these findings represent changes in measured performance rather than confirmed enhancement of physiological muscular strength, because familiarization and other measurement effects may have contributed. Gut-microbiota alpha diversity remained largely stable, apart from an FDR-significant reduction in the ACE index in the normal-weight group, while selected taxon-level changes indicated directional microbial remodeling during fasting. The exploratory sex-stratified findings further identified potentially informative subgroup patterns for future investigation. Collectively, this study provides an integrated characterization of short-term physiological and gut-microbial responses to complete water-only fasting and structured refeeding, and supports larger controlled studies to determine the reproducibility, duration, and clinical relevance of these responses.

## Data Availability

The raw data supporting the conclusions of this article will be made available by the authors, without undue reservation.

## Footnotes

1
http://www.chictr.org.cn/index.aspx

2
https://cnsknowall.com
