# Correction: The impact of short-term intensive fasting on physical health and gut microbiota in obese individuals

**DOI:** 10.3389/fnut.2026.1985899

**Published:** 2026-09-11

**Authors:** Feng Ji, Shiya Wang, Li Wang, Yixuan Zhao, Jiabin Zhong, Ruohan Wang, Jianing Qu, Yunhang Lu, Na Yuan, Qing Zhang

**Affiliations:** 1 School of Physical Education and Sports Science, Soochow University, Suzhou, China; 2 Changzhou Tourism and Commerce Higher Vocational School, Changzhou, Jiangsu, China; 3 Department of Community Nursing, Soochow University, Suzhou, China; 4 Hematology Center of Cyrus Tang Medical Institute, Soochow University, Suzhou, China; 5 Institute of Health Futures, Soochow University, Suzhou, China

**Keywords:** cardiovascular health, gut microbiome, obesity, physical health, short-term intensive fasting

The funder Soochow University (Grant No. AS10602725) was erroneously omitted from the Funding statement.

The correct **Funding** statement reads: “The author(s) declared that financial support was received for the conduct and/or publication of this work. This study was funded by Soochow University, Suzhou, China (Grant No. AS10602725) under the Humanities and Social Sciences Research (Applied Disciplines) Project: “Comprehensive Research on Health Management throughout the Life Cycle.” Additional support was provided by the National Natural Science Foundation of China (Grant No. 82470165, awarded to NY).”

In the **Acknowledgments**, “Yaozhong Hu” was misspelled as “Yaozhong Wu”.

The correct **Acknowledgments** statement reads: “We thank all the volunteers participating in this short-term intensive fasting study and the campus Hospital of Soochow University for providing the facilities for the collection of blood samples and taking physical measurements. We further thank Yaozhong Hu and his team for their advice on the short-term intensive fasting protocol.”

The original version of this article has been updated.

